# Generalization vs. Specificity: In Which Cases Should a Clinic Train its Own Segmentation Models?

**DOI:** 10.3389/fonc.2020.00675

**Published:** 2020-05-14

**Authors:** Jan Schreier, Francesca Attanasi, Hannu Laaksonen

**Affiliations:** Varian Medical Systems (United States), Palo Alto, CA, United States

**Keywords:** deep learning, segmentation, radiotherapy planning, neural network, generalizability

## Abstract

As artificial intelligence for image segmentation becomes increasingly available, the question whether these solutions generalize between different hospitals and geographies arises. The present study addresses this question by comparing multi-institutional models to site-specific models. Using CT data sets from four clinics for organs-at-risk of the female breast, female pelvis and male pelvis, we differentiate between the effect from population differences and differences in clinical practice. Our study, thus, provides guidelines to hospitals, in which case the training of a custom, hospital-specific deep neural network is to be advised and when a network provided by a third-party can be used. The results show that for the organs of the female pelvis and the heart the segmentation quality is influenced solely on bases of the training set size, while the patient population variability affects the female breast segmentation quality above the effect of the training set size. In the comparison of site-specific contours on the male pelvis, we see that for a sufficiently large data set size, a custom, hospital-specific model outperforms a multi-institutional one on some of the organs. However, for small hospital-specific data sets a multi-institutional model provides the better segmentation quality.

## Introduction

Radiotherapy is a common treatment modality for breast cancer as well as pelvic cancer types. During the planning process of the treatment, the target structure(s) as well as surrounding organs-at-risk (OAR) need to be segmented. This is a time-consuming task, which takes, for example, for a breast cancer case on average 31 min (1).

Previously, heuristic automatic segmentation algorithms have been developed to decrease the contouring effort. These algorithms are based on for example water shedding (2), thresholding (3) and region growing (4). Additionally, convolutional filters have been used to segment breast segments on MR images (5). In contrast to these heuristic algorithms, atlas-based methods have been developed for propagating the structures from a reference patient to a specific target patient (6, 7).

Recently, machine learning techniques such as random forests (8) and neural networks have emerged to prominence in medical image segmentation. More specifically, deep learning as an advanced machine learning algorithm has been frequently applied to similar problems (9). For example, fully convolutional neural networks have been used to segment organs in the head and neck region (10), in the thorax (11), abdomen (12) and pelvis (13, 14).

One common challenge in any automatic segmentation approach is the inter-observer variability (6). This inter-site variability leads to differing performances of the same algorithm among different hospitals (15). A study for a deep neural network on MRI showed that a multi-site model is more robust to unseen data than a single-site model (16). However, the differences between different MR scanners and acquisition protocols might explain part of the difference (17).

This study first compares the performance of a multi-site, single-site, and third-party model based on the different patient populations of various hospitals in different geographies. Here, the structures are created by the same group of experts to mitigate differing contouring practices. Finally, we compare the performance of the three different types of models on the male pelvis with the contours being created by the respective hospitals. Thus, the effect of the differences in contouring practice are visible.

## Methods

### Data

#### Female Breast

CT datasets from the databases of four institutions were selected for this study, one from North America (Clinic B) with 64 patients and three from Europe (Clinic A, C, and D) with 83, 86, and 47 patients respectively. The completed dataset consisted of heterogeneous CT breast scans to ensure balance with respect to diagnosis, age and body mass index. All patients were scanned in the supine position and immobilized either with breast boards (flat or low-angled) or vacuum cushions. Patients from Clinic B, C, and D were scanned with both arms up instead of the traditional one arm up used in Clinic B. All OARs were delineated according to RTOG guidelines (18) by three experienced dosimetrist and comprised left breast, right breast and heart. A summary of the data set together with the average volumes for each structure per clinic can be seen in Table 1.

**Table 1:** Displayed is information for each of the clinics of the female breast data set. The volumes are in cubic centimeters.

**Clinic**	**Geography**	**#Patients**	**#Scans**	**Volume Left Breast**	**Volume Right Breast**	**Volume Heart**
Clinic A	UK	83	83	1200 ± 556	1214 ± 566	654 ± 101
Clinic B	US	64	64	1240 ± 559	1271 ± 586	680 ± 103
Clinic C	Italy	86	86	808 ± 449	806 ± 446	597 ± 77
Clinic D	France	47	47	947 ± 461	958 ± 455	626 ± 101

#### Female Pelvis

The dataset for the female pelvis contains both CT, CBCT, and generated pseudo CBCT images from four different clinics. Clinic A and B are located in Europe with 79 and 298 scans from 14 and 33 patients respectively. Clinic C is located in India with 84 scans from 7 patients and Clinic D is located in North America with 92 CT scans and from these 92 generated CBCT scans. Clinic A, B, and C contain one to two CT scans and between one and 20 CBCT per patient. While some CT images contained structures from the original hospital, the CBCT scans did not have contours created by the clinics. Therefore, all contours have been either curated or redrawn by a group of five experienced dosimetrists. A summary of the data set together with the average volumes for each structure per clinic can be seen in Table 2.

**Table 2:** Displayed is information for each of the clinics of the female pelvis data set. The volumes are in cubic centimeters.

**Clinic**	**Geography**	**#Patients**	**#Scans**	**Volume Bladder**	**Volume Rectum**	**Volume Uterus**
Clinic A	Europe	79	298	226 ± 310	62 ± 34	114 ± 70
Clinic B	Europe	14	33	165 ± 127	51 ± 23	150 ± 148
Clinic C	India	7	84	278 ± 130	47 ± 22	108 ± 49
Clinic D	North America	92	184	115 ± 83	62 ± 36	179 ± 117

#### Male Pelvis

The dataset for the male pelvis contains CT images with structures for the bladder, rectum, prostate and seminal vesicle from four different clinics. Clinic A, C, and D are located in North America with 76, 25, and 55 patients respectively. Clinic B is located in Europe and provided 20 patients. For each patient, there is one 3D CT image with structures in the dataset. The structures are provided by the hospitals and are used unmodified with the exception of the seminal vesicles. Here, the original structure covered only the proximal part and was extended to cover the full organ. A summary of the data set together with the average volumes for each structure per clinic can be seen in Table 3.

**Table 3:** Displayed is information for each of the clinics of the male pelvis data set. The volumes are in cubic centimeters.

**Clinic**	**Geography**	**#Patients**	**#Scans**	**Volume Bladder**	**Volume Prostate**	**Volume Rectum**	**Volume Seminal Vesicle**
Clinic A	North America	76	76	389 ± 193	47 ± 21	80 ± 39	12 ± 4
Clinic B	Europe	20	20	294 ± 207	54 ± 21	71 ± 33	17 ± 6
Clinic C	North America	25	25	165 ± 106	43 ± 12	64 ± 19	9 ± 4
Clinic D	North America	55	55	256 ± 182	47 ± 20	52 ± 15	15 ± 6

### Architecture and Training

The BibNet architecture is used as its performance has been proven previously both for segmentation of female breast and heart on CT images (19) and for segmentation of OARs in the male pelvic region on CT and CBCT (15). For both models parametric rectified linear unit layers (PRelU) and dropout layers with a dropout rate of 0.5 are used.

The female breast model is trained on patches of size 256 × 256 × 32. During each training, 8 models were random initialized and the best performing one trained until 40,000 iterations. The Jaccard loss is used as a loss function for all models.

For the female and male pelvis the model is trained on patches of size 192 × 192 × 32. During each training, eight models are random initialized and the best performing one trained until 25,000 iterations. In most cases, the Jaccard loss is used as a training loss. However, in the case of a mode breakdown, that is to say if one structure is not contoured at the end of the training, the loss is switched to a Tversky loss with α = 2 and β = 0.5. Through this, false negatives are weighted more strongly than false positives, which circumvents the mode break down.

### Experiments

For all anatomical sites, a multi-site model is trained using training data from all clinics. Further, for each clinic a single-site model is trained using only its own data and a “third-party” is trained using all but its own data. To increase the statistical significance of the results, 5-fold cross validation is used for the single-site and the multi-site model. Additionally, a multi-site model is trained using only subsets of the training cases, while maintaining the same splits in test, validation and train set. As 5-fold cross validation is used in the before described experiments, each patient is once used in the test set, while not being present in validation or training.

For the size test on the breast data set the first split is trained using different sizes of 25, 50, 75, 100, 150, and 200 patients. This is due to the long training time for the breast models (~3 days/model on a Nvidia K80). On the female pelvis region 5-fold cross validation is used. The pelvic model is trained using 5 (38 ± 13), 10 (41 ± 10), 25 (115 ± 12), 50 (208 ± 57), and 75 (327 ± 18) patients, and training scans respectively. On the male pelvis, the sizes 10, 25, 50, 75, and 100 each one model is trained using the first training split of the above mentioned cross-validation.

### Evaluation

For the comparison between the multi-site and single-site models as well as for the difference between image-specific and multi-site model and position-specific and multi-site model a paired *t*-test is performed with the critical value set to 5%.

## Results

### Female Breast

**Figure 1:**
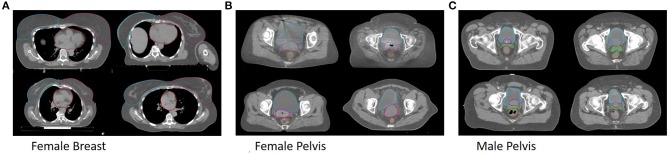
Shown are segmentations of the general models (dark) and the ground truth (bright) for example patients from Clinic A (top left), Clinic B (top right), Clinic C (bottom left) and Clinic D (bottom right) for the female breast **(A)**, the female pelvis **(B)**, the male pelvis **(C)**.

All trained models are able to segment both breast and the heart. Example segmentations from the multi-site model together with the ground truth for one patient per clinic can be seen in Figure 1A.

**Figure 2:**
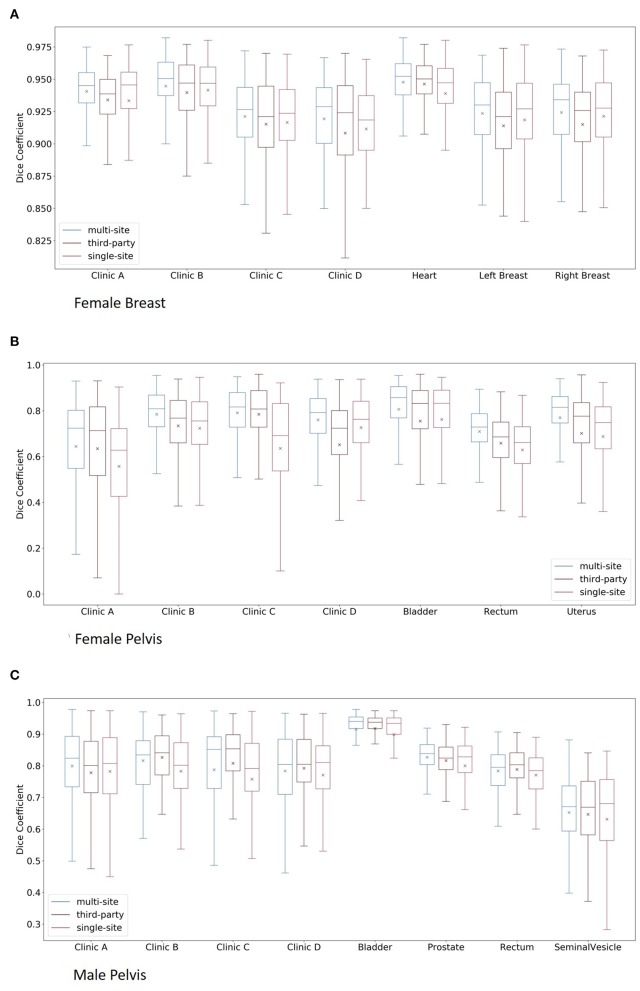
Boxplots of the multi-site, third-party and single-site model per clinic and per organ for the female breast **(A)**, the female pelvis **(B)**, and the male pelvis **(C)**. Here, for the plots per clinic, all organs are combined, whereas for the plots per organs, all clinics are combined.

The box plots of the comparisons are shown in Figure 2A. For clinics, all of the organs are combined in the plot, and for the organs, all of the clinics are combined. The median dice scores together with the results of the statistical tests can be found in Table 4.

**Table 4:** The median dice score with standard deviation for the organs of the female breast and the three different model types.

	**Clinic A**			**Clinic B**			**Clinic C**			**Clinic D**		
	**Multi-site**	**Third-party**	**Single-site**	**Multi-site**	**Third-party**	**Single-site**	**Multi-site**	**Third-party**	**Single-site**	**Multi-site**	**Third-party**	**Single-site**
Left breast	0.942 ± 0.024	0.932 ± 0.024	**0.947** ± 0.057	**0.941** ± 0.025	0.940 ± 0.031	0.935 ± 0.039	0.913 ± 0.029	0.905 ± 0.033	**0.914** ± 0.035	**0.917** ± 0.043	0.905 ± 0.051	0.903 ± 0.042
Right breast	0.945 ± 0.022	0.937 ± 0.023	**0.949** ± 0.025	**0.942** ± 0.023	0.934 ± 0.028	0.939 ± 0.024	**0.914** ± 0.034	0.909 ± 0.037	0.913 ± 0.041	**0.915** ± 0.036	0.909 ± 0.066	0.908 ± 0.038
Heart	**0.949** ± 0.017	0.947 ± 0.018	0.943 ± 0.105	**0.964** ± 0.017	0.963 ± 0.011	0.963 ± 0.015	**0.949** ± 0.020	**0.949** ± 0.021	0.946 ± 0.022	0.946 ± 0.020	**0.947** ± 0.026	0.936 ± 0.026
Combined	0.945 ± 0.022	0.939 ± 0.023	**0.946** ± 0.070	**0.951** ± 0.025	0.947 ± 0.029	0.947 ± 0.031	**0.927** ± 0.032	0.921 ± 0.037	0.924 ± 0.038	**0.929** ± 0.038	0.924 ± 0.056	0.918 ± 0.039

*For each clinic, a multi-site model, which was trained using data from all four clinics, a third-party model, which is trained on data from all clinics but the one it is evaluated on, and a single-site model is shown. In bold is the best performing model per organ and clinic. The cell is shaded in gold if the difference between the best and the second-best model per category is statistically significant and in grey if the difference between the best and the worst model per category is statistically significant*.

Overall the multi-site model performs statistically significantly better for Clinic B, C, and D than both the third-party and the single-site model. On Clinic A, the single-site model performs best, while being statistically significantly compared to the third-party model but not the multi-site model.

With respect to the heart, the multi-site model performs best for all except Clinic D, where the third-party model performs better. For the latter, the difference is statistically significant toward the single-site model but not the multi-site model.

Concerning the breasts, the single-site model performs best for Clinic A and on the left breast on Clinic C. For these cases, the difference between the single-site and the third-party model is statistically significant but not toward the multi-site model. On the other hand, the multi-site model performs better than the other two on Clinic B and D and for the right breast of Clinic C. The differences to the third-party model are statistically significant for Clinic B and C and toward the single-site model for the left breast on Clinic B and the right breast on Clinic C. The differences in Clinic D are not statistically significant. On Clinic B, the single-site model performs better than the third-party model on the right breast but worse on the left breast. Whereas, on Clinic D, which has the smallest data set, the third-party model performs slightly better but not statistically significant than the single-site model.

**Figure 3:**
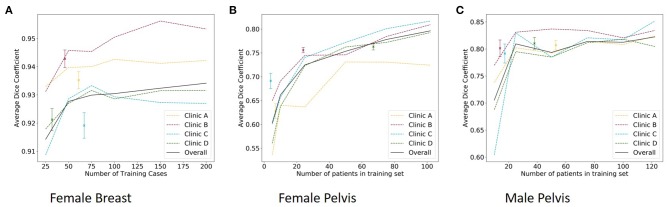
The median dice score of single-site model with standard deviation (dots) and the results of the size test per clinic and overall (lines) for the female breast **(A)**, the female pelvis **(B)**, and the male pelvis **(C)**.

The results of the size test can be seen in Figure 3A.

### Female Pelvis

All trained models are able to segment bladder, rectum and uterus. Example segmentations from the multi-site model together with the ground truth for one patient per clinic can be seen in Figure 1B.

The results of the multi-site, third-party and single-site model can be found in Table 5. Further, a box plot of the results is shown in Figure 2B.

**Table 5:** The median dice score with standard deviation for the organs of the female pelvis and the three different model types.

	**Clinic A**			**Clinic B**			**Clinic C**			**Clinic D**		
	**multi-site**	**Third-party**	**Single-site**	**multi-site**	**Third-party**	**Single-site**	**multi-site**	**Third-party**	**Single-site**	**multi-site**	**Third-party**	**Single-site**
Bladder	**0.791** ± 0.249	0.726 ± 0.277	0.663 ± 0.266	**0.873** ± 0.113	0.850 ± 0.150	0.861 ± 0.188	**0.908** ± 0.056	0.904 ± 0.069	0.869 ± 0.181	**0.797** ± 0.171	0.768 ± 0.260	0.789 ± 0.144
Rectum	**0.702** ± 0.172	0.693 ± 0.139	0.631 ± 0.148	**0.733** ± 0.110	0.667 ± 0.127	0.682 ± 0.130	0.720 ± 0.107	**0.726** ± 0.120	0.583 ± 0.216	**0.738** ± 0.093	0.703 ± 0.144	0.686 ± 0.162
Uterus	0.648 ± 0.237	**0.726** ± 0.280	0.514 ± 0.247	**0.821** ± 0.103	0.789 ± 0.118	0.746 ± 0.140	0.808 ± 0.158	**0.813** ± 0.170	0.665 ± 0.255	**0.840** ± 0.136	0.730 ± 0.278	0.816 ± 0.137
Combined	**0.724** ± 0.229	0.714 ± 0.244	0.628 ± 0.233	**0.809** ± 0.120	0.768 ± 0.150	0.756 ± 0.165	**0.817** ± 0.134	0.808 ± 0.147	0.691 ± 0.253	**0.793** ± 0.141	0.724 ± 0.236	0.763 ± 0.159

*For each clinic, a multi-site model, which was trained using data from all four clinics, a third-party model, which is trained on data from all clinics but the one it is evaluated on, and a single-site model is shown. In bold is the best performing model per organ and clinic. The cell is shaded in gold if the difference between the best and the second-best model per category is statistically significant and in grey if the difference between the best and the worst model per category is statistically significant*.

The mean dice score for the multi-site and single-site model respectively are 0.81 and 0.76 for the bladder, 0.71 and 0.63 for the rectum and 0.77 and 0.69 for the uterus. The average dice score per clinic over all three organs for the multi-site and single-site model respectively are 0.64 and 0.56 for Clinic A, 0.79 and 0.72 for Clinic B, 0.79 and 0.63 for Clinic C, and 0.76 and 0.73 for Clinic D. The differences between the multi-site and the single-site model are statistically significant for all clinics and organs.

Overall, the multi-site model performs best for all four clinics with the difference being statistically significant toward the single-site model in all cases and toward the third-party model for Clinic B and D. The multi-site model performs statistically significantly better than the single-site and third-party model for all organs compared separately on Clinic B and D as well as for the Bladder on Clinic A. Even though the multi-site model performs best on the rectum for Clinic I and the bladder for clinic C, this difference is only statistically significant compared to the single-site model but not the third-party model. On Clinic A and C the third-party model performs best on the uterus and on clinic B on the rectum, with the differences being statistically significant only compared to the single-site model.

The results of the size test can be found in Figure 3B.

### Male Pelvis

All trained models are able to segment bladder, prostate, rectum and seminal vesicle. Example segmentations from the multi-site model together with the ground truth for one patient per clinic can be seen in Figure 1C.

The results of the multi-site, third-party and single-site model can be found in Table 6. Further, a box plot of the results is shown in Figure 2C.

**Table 6:** The median dice score with standard deviation for the organs of the male pelvis and the three different model types.

	**Clinic A**			**Clinic B**			**Clinic C**			**Clinic D**		
	**multi-site**	**Third-party**	**Single-site**	**multi-site**	**Third-party**	**Single-site**	**multi-site**	**Third-party**	**Single-site**	**multi-site**	**Third-party**	**Single-site**
Bladder	**0.948** ± 0.091	0.945 ± 0.134	0.944 ± 0.161	0.927 ± 0.02	0.931 ± 0.021	**0.932** ± 0.034	0.921 ± 0.032	**0.924** ± 0.036	0.900 ± 0.056	**0.928** ± 0.178	0.925 ± 0.079	0.917 ± 0.142
Prostate	**0.837** ± 0.061	0.808 ± 0.056	0.834 ± 0.087	0.838 ± 0.034	**0.842** ± 0.047	0.813 ± 0.061	**0.868** ± 0.066	0.867 ± 0.052	0.857 ± 0.125	**0.826** ± 0.064	0.819 ± 0.070	0.824 ± 0.146
Rectum	**0.799** ± 0.064	0.790 ± 0.074	0.778 ± 0.076	0.799 ± 0.078	**0.839** ± 0.071	0.775 ± 0.073	0.805 ± 0.069	**0.842** ± 0.078	0.775 ± 0.070	0.775 ± 0.074	0.787 ± 0.078	**0.795** ± 0.071
Seminal vesicle	0.663 ± 0.125	0.630 ± 0.140	**0.690** ± 0.144	0.723 ± 0.077	**0.755** ± 0.086	0.721 ± 0.158	0.614 ± 0.177	**0.672** ± 0.135	0.631 ± 0.201	**0.681** ± 0.101	0.679 ± 0.116	0.662 ± 0.174
Combined	**0.825** ± 0.123	0.804 ± 0.138	0.811 ± 0.132	0.834 ± 0.096	**0.841** ± 0.093	0.801 ± 0.135	0.852 ± 0.165	**0.854** ± 0.139	0.792 ± 0.175	0.814 ± 0.118	0.809 ± 0.115	**0.816** ± 0.126

*For each clinic, a multi-site model, which was trained using data from all four clinics, a third-party model, which is trained on data from all clinics but the one it is evaluated on, and a single-site model is shown. In bold is the best performing model per organ and clinic. The cell is shaded in gold if the difference between the best and the second-best model per category is statistically significant and in grey if the difference between the best and the worst model per category is statistically significant*.

The results of the size test can be found in Figure 3C.

The multi-site model provides overall the best performance for Clinic A, with the difference being statistically significant only toward the third-party model. On Clinic B and C, the third-party model, which has here the largest training set size of 140 and 136 respectively, performs best. The difference is statistically significant toward the single-site model. For Clinic D, the single-site model performs best. When comparing the third-party and the single-site model, we can see that on Clinic A, the prostate and the seminal vesicles are contoured better by the single-site model. On Clinic D, the rectum and prostate are contoured better by the single-site model. On Clinic B, the single-site model performs better on the bladder, while not being statistically significant. For the remaining Clinics and organs, the third-party model performs better than the single-site model.

## Discussion

The population-based experiment on the female breast and female pelvis show that there seems to be no advantage for a site-specific model for the organs of the female pelvis and the heart. However, for the female breast there is an advantage of a site-specific model compared to a third-party model if the training set is sufficiently large. This could be explained through the larger inter-population variation of the female breast compared to the internal organs of the female pelvis and the heart. As the shape and size of the breasts correlates with factors such as geography (20) and body mass index (21) of the patient population, the breasts of patients from clinics, which are in different geographies, differ. This impact of geography is observed in the differences of the average breast volumes in this study, with the patients from UK and US having on average a larger breast volume than the patients from Italy and France. This correlates with the differing average body mass indices in the respective countries, with US and UK having larger average body mass indices than France and Italy (22). Therefore, a model, which has not been trained with patients of a certain geography, suffers from an inferior quality compared to a site-specific model, which has seen patients from its geography. This study suggests that this is the case even if the training set size is smaller. In contrast to this, the anatomy of the heart and the organs of the female pelvis are less anatomically variable and the anatomical boundaries are better defined than for the breast.

The experiment based on different clinical practice on the male pelvis relies on the conclusion drawn from the population-based experiment. There, we could see that for internal organs the population difference does not play a significant role. Therefore, the differences between the different model types in the male pelvis experiment are mainly based on the different training set sizes and differing clinical practices. We can see that for the two smallest hospitals, the site-specific models perform worse when compared to both the third-party and the multi-site model. This is most likely due to their small training set sizes of 14 and 17 patients. In the other two hospitals, we can see that overall the multi-site model is either better (Clinic A) or equivalent (Clinic D) to the site-specific model. When comparing per structure the site-specific to the third-party model, we can see that for the Clinic A the prostate and seminal vesicles perform worse. For Clinic D, the rectum performs worse on the multi-site and third-party model compared to the single-site model. This might be due to a varying contouring practice of this hospital, e.g., a different inferior and superior end of the rectum. For both Clinic A and D, the third-party model is outperformed overall by the site-specific model. One might note here that the site-specific models have less data available for training than the third-party model and increasing the data set size might increase the described effect.

Our experiments show that the multi-site model performs equally or better than a model trained specifically for each clinic. For the female breast, this is true even when comparing models trained with similar training set sizes. For the female pelvis, the difference is ambiguous when comparing equal number of training patients. This result is influenced by the varying number of scans per patient. When considering the average number of training scans in each split, there is no clear difference between the single-site and the multi-site model. For the male pelvis, the site-specific models perform better than the multi-site model, when the number of training patients is equal. Thus, the improved performance previously mentioned seemed to be due to the increase number of training cases.

For the female pelvis on Clinic D, the third-party model performs worse than a single-site model. Here, the difference might be caused through the lower number of CT scans in the training set of the third-party model. The comparison between the third-party model and the multi-site model showed that the multi-site model performs better for all clinics on the bladder and for all but one clinic on the rectum. Interestingly, the third-party model performs better on the uterus than the multi-site model for Clinic A–C but worse than the multi-site and the single-site model for Clinic D. The reason for this could be similar to the uterus result on CBCT: During the training of the multi-site model, 30% of the Clinic D, which contains 61% of the CT scans, is excluded from the training set. Therefore, less accurate ground truth for uterus delineation exists in the multi-site model compared to the third-party model, which uses the full data set from Clinic D as training and validation set. The opposite holds true for the third-party model for Clinic D: Here, only 39% of the CT scans can be used in training and the majority of the training set contains CBCT scans. Thus, the performance on Clinic D is deteriorated such that both the multi-site model and the single-site model, which had access to less scans, perform better than the third-party model.

On the male pelvis, the multi-site model performs best for Clinic A, the third-party for Clinic B and C and the results are not statistically significant for Clinic D. This result suggests that overall the performance is dictated by the training set size and not by the specificity toward one clinics practice. While the training set for the multi-site model contains on average 122 patients, the third-party and the single-site training set for Clinic A contain only 90 and 53 patients, respectively. In contrast to this, the training set for Clinic B (Clinic C) for the third-party contains 140 (136) patients, and for the single-site model 14 (17), respectively. For Clinic D the data for the third-party model contains 109 patients and for the single-site model 38 patients.

## Conclusion

The study suggests that a third-party model can be readily applied to organs with low inter-hospital variability; in our data sets the organs of female pelvis as well as the heart were examples where the contouring practices and anatomies were consistent. However, the performance of a third-party model is poorer compared to both a single-site and a multi-site model on the breasts as here a higher variability in the patient anatomy exists. Further, if a clinic possesses a sufficient number of training cases a site-specific model can outperform a third-party model in the case if the clinical practice differs. Examples for this are the boundary of the prostate and seminal vesicle as well as the definition of the superior and inferior end of the rectum.

## Data Availability Statement

The datasets generated for this study will not be made publicly available. The data is proprietary.

## Ethics Statement

Ethical review and approval and written informed consent from patients were not required for the study on human participants in accordance with the local legislation and institutional requirements.

## Author Contributions

JS initialized the work, executed the experiments, and wrote the majority of the manuscript. FA supervised the clinical collaboration and data curation. HL supervised the work. All authors contributed to the manuscript.

## Conflict of Interest

All authors were employed by the company Varian Medical Systems, Palo Alto.
